# Mechanistic or mammalian target of rapamycin (mTOR) may determine robustness in young male mice at the cost of accelerated aging

**DOI:** 10.18632/aging.100528

**Published:** 2012-12-21

**Authors:** Olga V. Leontieva, Geraldine M. Paszkiewicz, Mikhail V. Blagosklonny

**Affiliations:** Department of Cell Stress Biology, Roswell Park Cancer Institute, BLSC, L3-312, Elm and Carlton Streets, Buffalo, NY 14263, USA

**Keywords:** MTOR, mTOR Target of rapamycin, growth, aging

## Abstract

Males, who are bigger and stronger than females, die younger in most species from flies to mammals including humans. Cellular mass growth is driven in part by mTOR (Target of Rapamycin). When developmental growth is completed, then, instead of growth, mTOR drives aging, manifested by increased cellular functions, such as hyper-secretion by fibroblasts, thus altering homeostasis, leading to age-related diseases and death. We hypothesize that MTOR activity is elevated in male mice compared with females. Noteworthy, 6 months old males were 28 % heavier than females. Also levels of phosphorylated S6 (pS6) and phospho-AKT (p-AKT, Ser 473), markers of the mTOR activity, were higher in male organs tested. Levels of pS6 were highly variable among mice and correlated with body weight and p-AKT. With age, the difference between levels of pS6 between sexes tended to minimize, albeit males still had hyperactive mTOR. Unlike fasting, the intraperitoneal (i.p.) administration of rapamycin eliminated pS6 in all organs of all females measured by immunoblotting and immunohistochemistry without affecting p-AKT and blood insulin. Although i.p. rapamycin dramatically decreased levels of pS6 in males too, it was still detectable by immunoblotting upon longer exposure. Our study demonstrated that both tissue p-AKT and pS6 were higher in young males than young females and were associated with increased body weight and insulin. These data can explain larger body size and faster aging in males. Our data suggest higher efficacy of rapamycin compared to fasting. Higher sensitivity of females to rapamycin may explain more pronounced life extension by rapamycin observed in females compared to males in several studies.

## INTRODUCTION

One of the most long-standing mysteries of gerontology is that the females of most species live longer than the males [1-12]. Not only most mammals but also women of different nations and at most historical periods live longer [2]. Ironically, it may seem that males do not age faster but simply are weaker at any age. In fact, the mortality rate is higher in young males and teenagers too. Importantly, however, old males die from age-related diseases, whereas young males mostly die from risky behavior and physical competition with each other. While risky competition increases chances of mating and offspring, this simultaneously results in high accidental mortality (from fights) and males die young. There is no reason for them to be naturally selected for slower aging. Therefore, animals with a high accidental death rate tend to age faster. It is exceptionally important for such males early in life to be bigger and stronger (even at the cost of accelerated aging).

Growth is driven by the mammalian Target of Rapamycin (mTOR) pathway [13-23]. (Note: mTOR is also very recently renamed as MechanisticTarget of Rapamycin (MTOR), so we will continue to use mTOR in this paper). TOR is conserved from yeast to mammals, including humans [24-26]. The mTOR pathway stimulates protein synthesis and many cellular functions, including secretion of mitogens, insulin and cytokines, which remind the senescent phenotype [27-30]. In postmitotic non-dividing cells, instead of size growth mTOR drives aging [31-37]. mTOR can convert reversible quiescence into irreversible senescence (geroconversion) [27, 38-43]. TOR pathway is involved in aging from yeast to worms to mammals [44-59] as well as in age-related diseases in mammals [15, 60-71].

Rapamycin suppresses cellular senescence [27, 31-36, 38-41, 72-80] and prolongs life span and health span in diverse species [45, 46, 50, 67, 71, 81-91]. By inhibiting TOR [36, 77, 92-94] p53 can suppress geroconversion [76, 95-98] and affect lifespan [99], [100] and diseases of aging [101]. Also some drugs, other than rapalogs, can alter lifespan by targeting the mTOR pathway [102-109]. Therefore, TOR emerges as a reasonable candidate gene that may determine both growth and aging.

In brief, early in life, TOR drives growth, robustness and reproduction, while causing aging and age-related diseases later in life [110-113]. This example of antagonistic pleiotropy is in line with the evolutionary theory [110]. We speculate that aging as a continuation of growth driven by the same mTOR pathway, leading to aging and diseases of aging culminating in organismal damage and death. The mTOR pathway is extremely complex [114-124]. It is stimulated by nutrients (food), insulin, insulin-like growth factor 1 (IGF-1), testosterone, oxygen, and pro-inflammatory cytokines [39, 55, 62, 114-130]. The TOR kinase forms 2 complexes: mTORC1 and mTORC2 [121]. mTORC1 is rapamycin-sensitive. This complex is characterized by the classic features of mTOR as a nutrient/energy/redox sensor, which controls protein synthesis and growth. Most importantly it promotes geroconversion (conversion from resting state to senescent phenotype) that is partially suppressed by rapamycin. Its most studied target is S6 kinase, which phosphorylates S6 and rapamycin prevents this phosphorylation. Therefore we used pS6, as a marker of mTORC1 activity, the most relevant to growth and aging. mTORC2 is rapamycin-insensitive. mTORC2 is a regulator of the cytoskeleton through its stimulation of F-actin stress fibers, paxillin, and protein kinase Cα (PKCα). mTORC2 phosphorylates the serine/threonine protein kinase Akt/PKB at a serine residue 473 (S473). Phosphorylation of the serine stimulates Akt phosphorylation at a threonine 308 residue by PDK1 and leads to full Akt activation [20, 116, 117, 121, 131-136].

In this study we used pS6 as a marker of mTORC1 activity - the major pro-aging pathway, and p-Akt S473 as a presumable marker of TORC2 activity, although it is also an activator of TOR, acting upstream of mTOR complexes.

In sum, mTOR may drive both growth and aging, associated with hyper-functions coupled with signal-resistance and malfunction, loss of homeostasis, leading to development of deadly diseases of aging such as cardiovascular and metabolic diseases, neuro-degeneration, cancer and organ atrophy or failure [65]. We hypothesize that males have a higher levels of mTOR activity, providing advantage (and bigger size) for young males even though accelerated aging and early death might follow.

## RESULTS

### Insulin and weight are higher in young male mice

**Figure 1:**
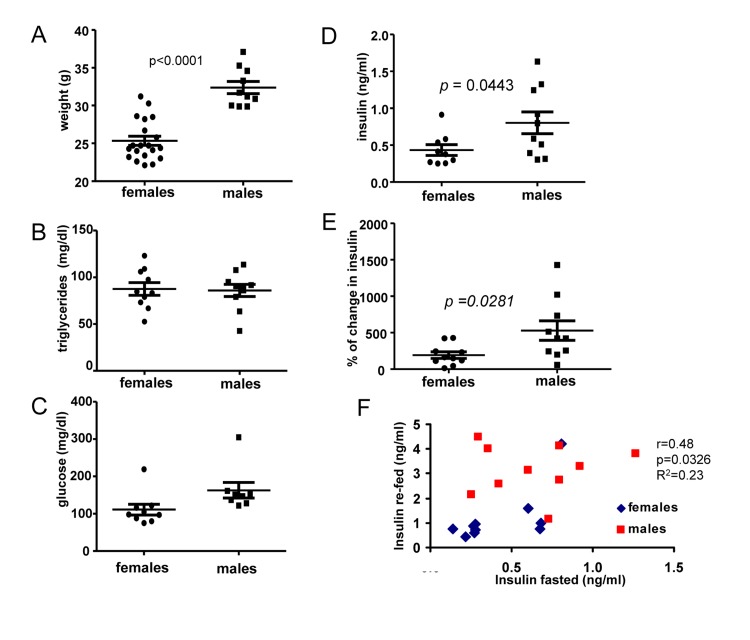
Metabolic profile of 6 months old males and females (**A**) Weight (grams) of 6 months old female and male mice. Data present mean ± SE. (**B**) Fasting serum triglyceride levels of females and males. Data are mean ± SE. (**C**) Fasting serum glucose levels in fasted blood of females and males. Data are mean ± SE. (**D**) Insulin serum levels in females and males. Data are mean ± SE. (**E**) Percent of increase in insulin levels in response to re-feeding after fasting by females and males. Data present mean ± SE. (**F**) Correlation between fasting insulin levels and levels of insulin 2 h after re-feeding. *r* – Pearson coefficient.

First, we compared 6 months old male and female mice. The most noticeable difference between males and females was body weight (Fig. 1A). At the age of 6 months, males were 28 % heavier than females. Females and males did not differ in levels of fasted triglycerides (Fig. 1B) and glucose (Fig. 1 C), as expected. Fasted insulin levels were slightly, but statistically significantly, increased in males (Fig. 1D). We also measured insulin response to re-feeding. Induction of insulin upon re-feeding was significantly higher in males (Fig. 1E). Moreover, levels of insulin after fasting correlated with higher levels of insulin after re-feeding (re-fed) and levels of both fasted and “re-fed” insulin were preferentially higher in males (Fig. 1F).

### The mTOR pathway is over-activated in 6 months old males

**Figure 2:**
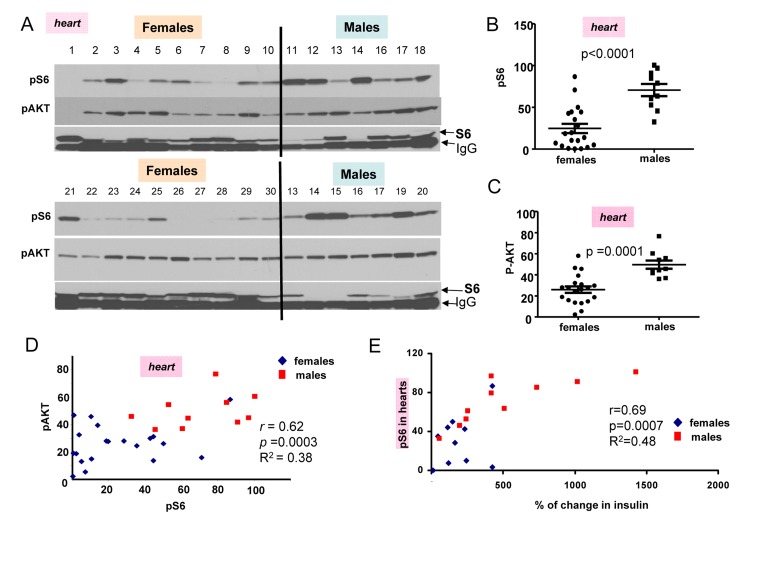
Comparison of pS6 and p-Akt levels in the hearts of 6 month old females and males (**A**) Immunoblot analysis of protein lysates from the hearts of 6 months old females and males, which were fed ad libitum, fasted overnight for blood collection and then re-fed for 2 hours. Numbers above blots represent individual mice. All mice, except numbers 21-30 underwent this schedule and were well fed before organ collection. In addition some females (21-30) received food ad libitum all the time (without transient fasting). Two conditions were considered as fed ad libitum (at least for 2 hours before sacrifice and organ collection). There was no difference in pS6 and Akt between two subgroups of mice (numbers 1-10 versus 21-30). Also there was no difference between levels of insulin and triglycerides in two sub-groups of females (Fig. 1S), confirming that they were of similar feeding status at the time of organ collection. Because of that we combined two female subgroups for further statistical analysis to increase statistical power to compare with males that were all similar re-fed for 2 hours as females. Now, all comparison of pS6 and p-AKT could be done between males and females as fed ad libitum for the last 2 hours. **Quantitative analysis of data shown in Figure 2A.** (**B**) Quantified intensities of phosphorylated S6 (pS6) signal in the hearts of female (n=20) and male (n=10) mice. Data are presented as mean ± SE. (**C**) Quantified intensities of p-AKT signal in the hearts of female (n=20) and male (n=10) mice. Data are presented as mean ± SE. (**D**) Correlation between levels of pS6 and p-AKT in the hearts. *r* – Pearson coefficient. (**E**) Correlation between levels of pS6 (in hearts) and an increase in insulin levels upon re-feeding in both females and males taken together.

In first series of experiments, blood was collected twice (after fasting and 2 hour after re-fed) and animals were sacrificed to measure pS6 and pAkt levels (Fig. 2 A). Levels of pS6 were variable, whereas levels of p-AKT were less variable between individual mice (individual mice were identified by numbers shown above each blot). (Note: Levels of total S6 (non-phosphorylated) were difficult to determine because S6 location on the blots is coincided with mouse immunoglobulin Gs, contaminating organs and recognizable by the secondary anti-mouse antibody.) However, as it is often observed in culture, pS6 coincided with disappearance of S6 (Fig. 2A).

**Figure 3:**
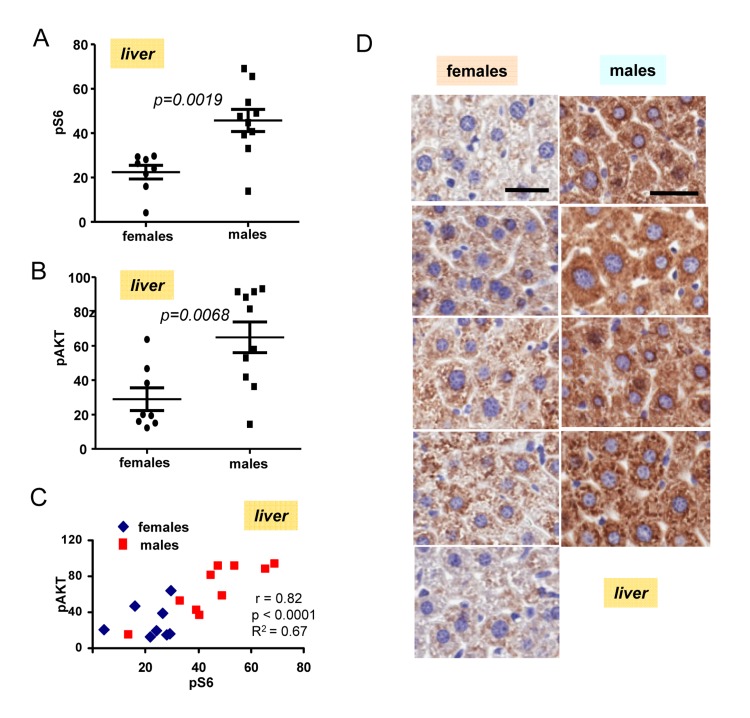
Levels of pS6 and pAKT in the livers of 6 months old females and males (**A**) Quantified intensities of pS6 signal in livers of females (n=8) and males 10). Data are mean ± SE. (**B**) Quantified intensities of p-Akt signal in livers of females (n=8) and males 10). Data are mean ± SE. (**C**) Correlation between levels of pS6 and p-AKT in livers. (**D**) Immunohistochemistry. pS6 in the livers of individual males and females. Mice were fasted overnight, then re-fed and sacrificed 2 h later. Bar – 30 μm.

The most important discovery was that levels of pS6 were significantly (p <0.0001) higher in male hearts (Fig. 2B). Similarly, levels of p-AKT were higher in males when measured in the hearts (significance p = 0.0001) (Fig. 2C). Importantly, levels of pS6 and p-AKT in the hearts strongly correlated in a combined group of all males and females taken together (high p-AKT corresponded to high pS6) (Fig. 2D). Also we found strong correlation between surge of insulin after re-feeding and levels of pS6 in the hearts (Fig. 2E). Furthermore, pS6 and p-AKT were elevated in male livers (Fig. 3). In male livers, levels of pS6 were several times higher and statistically significant in comparison to females (Fig. 3A). Similarly, levels of p-AKT were also statistically significantly higher in males (Fig. 3B). There was a strong correlation between the levels of p-AKT and pS6 in livers of all mice in this study (Fig. 3C). To confirm results obtained by immunoblotting we stained for pS6 sections of the livers from all the mice. Immunohistochemistry demonstrated that all individual male mice had elevated levels of pS6 compared with females (Fig. 3D). Noteworthy, pS6 was localized in the cytoplasm of hepatocytes. Levels of pS6 in females were so low compared with males, that it was possible to see staining as small cytoplasmic grains (Fig. 3D). Thus two different methods and two different organs demonstrated the same results.

### Effect of fasting and rapamycin injections in 10 months old mice

**Figure 4:**
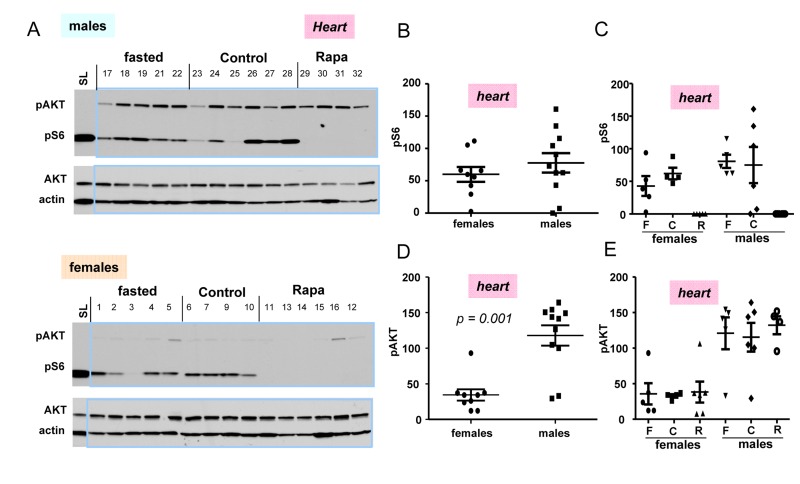
Levels of pS6 and p-AKT in the hearts of 10 months old mice: control, fasted, rapamycin (**A**) Immunoblot analysis of protein lysates from the hearts of females and males. Numbers above blots are individual mice in each group. Standard loading (SL) – 10 microgram lysates from cultured cells were loaded onto each upper and lower gels and blots were exposed to comparable intensities in SL lanes,. Fasted: mice were fasted for 16 h and sacrificed, control: mice were fed ad libitum, Rapa: “control” mice were treated with 1.5 mg/kg rapamycin (i.p.) 1 h before sacrifice. (**B, D**) – Quantified intensities of pS6 and p-Akt signal in hearts of females versus males. Female and male groups were comprised of all animals un-treated with rapamycin (n = 9 in female group and n = 11 in male group) regardless of feeding status. Data are mean ± SE. (**C**, **E**) Quantified intensities of pS6 and p-Akt signal in each sub-group. Mean ± SE in each subgroup separately: F – fasted; C – control; R – rapamycin-treated subgroup.

In next series of experiments, we subdivided 10 months old mice in 3 sub-groups: fasted, fed ad libitum (control), and control mice treated with rapamycin (Fig. 4). First, we noticed that the difference in p-AKT and pS6 in hearts between control and fasted females as well as between control and fasted males (Fig. 4A) were not significant (Fig. 4 C,E)). We combined two sub-groups of males (individuals with numbers 17-22 plus 23-28) into the male group and, similarly, two sub-groups of females (individuals with numbers 1-5 plus 6-10) into the female group (Fig. 4. B, D) to compare pS6 (Fig. 4 B) and p-AKT (Fig. 4 D) directly between two groups: females vs males (Fig. 4B, D).

(Note: rapamycin-treated mice were not included in such groups and were compared with non-treated groups separately in Fig. 4 C, E). There was a tendency to a higher pS6 in male hearts (Fig. 4 B); however a number of mice was likely to be too small to obtain statistical significance. P-AKT was statistically and dramatically over-expressed in the hearts of males compared with females (Fig. 4 D).

Most measurements were done using 18-well gel/blots. In order to compare levels of pS6 and p-AKT on different blots, produced at different times, with different antibody manufactures and different times of exposure, we decided to include cell culture lysates as loading/exposure controls in each gel. In Fig. 4A, we used 10 μg of the HT-p21-9 lysates used by us in previous studies. For tissues we used 30 ug of protein per well. We found that levels of pS6 in mice tissues were at least an order of magnitude lower than levels of pS6 in cultured cells (Fig. 4A). This result may reflect more “contact-inhibited” conditions in the organs and also less stimulation of the mTOR pathway in normal cells in the organism compared with cancer cells (HT-p21-9 cells). Comparison of levels of mTOR activation in the organism and cell culture will be the subject of separate investigation. Also interestingly, levels of p-AKT were much higher in animal organism than in cultured cells. Levels of p-AKT were significant higher in male hearts compared to females (Fig. 4C).

### Rapamycin decreased pS6 but did not affect p-AKT

While rapamycin decreased pS6 in male and female hearts; it did not affect levels of p-AKT (Fig. 4A, B, and C).

### Rapamycin did not completely inhibit pS6 in males compared to females

**Figure 5:**
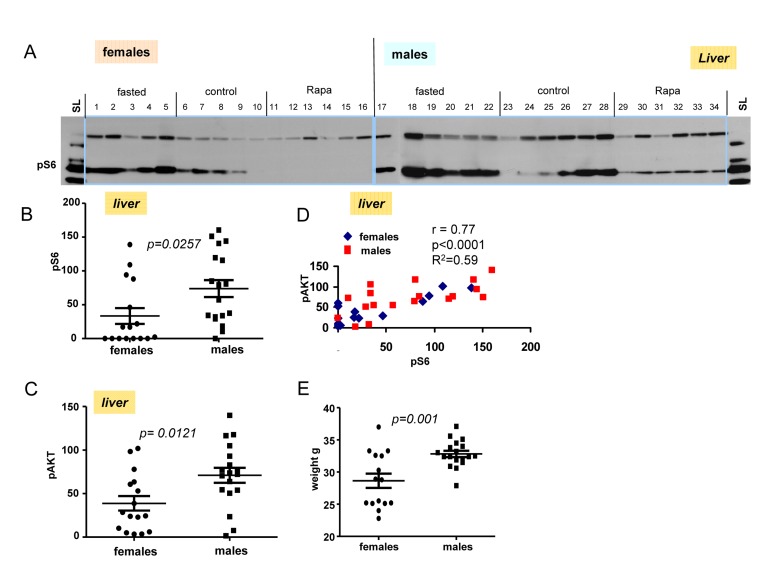
Levels of pS6 and p-AKT in the livers of 10 months old mice: control, fasted, rapamycin (**A**) Immunoblot analysis of protein lysates from livers of older (10 months old) females and males. Numbers indicate individual mice in each group. SL – standard loading,1 ug lysates from cultured cells was loaded onto each gel (left and right) and blots were over-exposed to obtain comparable intensities in standard loading lanes. (**B**) Quantified intensities of phosphorylated S6 (pS6) signal in livers of females and males. Left panel – female and male groups were comprised of all the animals from 3 groups (fasted, control and rapamycin) – n = 15 in female group and n = 18 in males group. Data are mean ± SE. (**C**) Quantified intensities of phosphorylated AKT (Ser 473) in livers of females and males. Data presented as described in legend **B**. (**D**) Correlation between levels of pS6 and p-AKT in livers from all the mice under study. (**E**) Comparison of older (~10 months old) female and male mice weights. Data present mean ± SE.

Next we performed immunoblot of tissues obtained from livers from fasted, control and rapamycin-treatedmales and females (Fig. 5A). Levels of pS6 and p-AKT in males were statistically higher then these levels in females (Fig. 5B and C). Using longer exposure, evidenced by the signal obtained from of loading control lysate (SL), we found that pS6 was detectable in the livers from males treated with rapamycin (Fig. 5A). Like in hearts (shown in Fig. 4), rapamycin also did not affect p-AKT in livers of both males and females (Fig. 5A).

**Figure 6:**
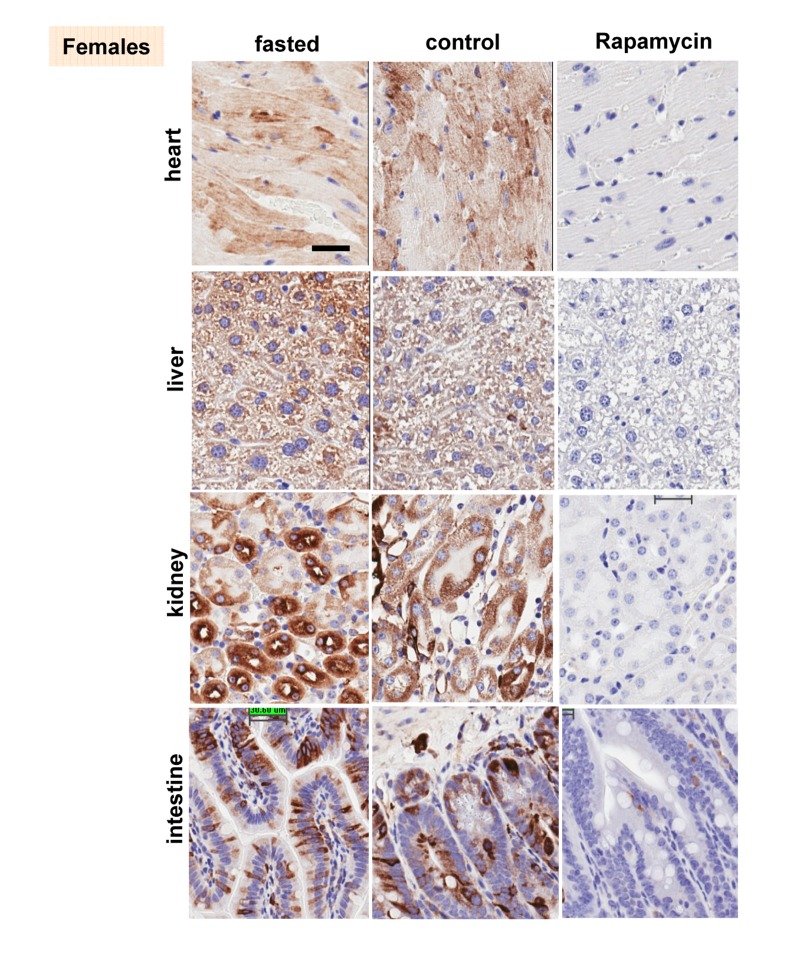
Females: Immunochemistry of pS6 in the organs pS6 in different organs of females (~ 10 months old). Fasted - mice were fasted overnight and sacrificed; control – mice received food ad libitum; rapamycin – mice received 1.5 mg/kg rapamycin i.p. and sacrificed 1 h later Bar. – 30 μm.

**Figure 7:**
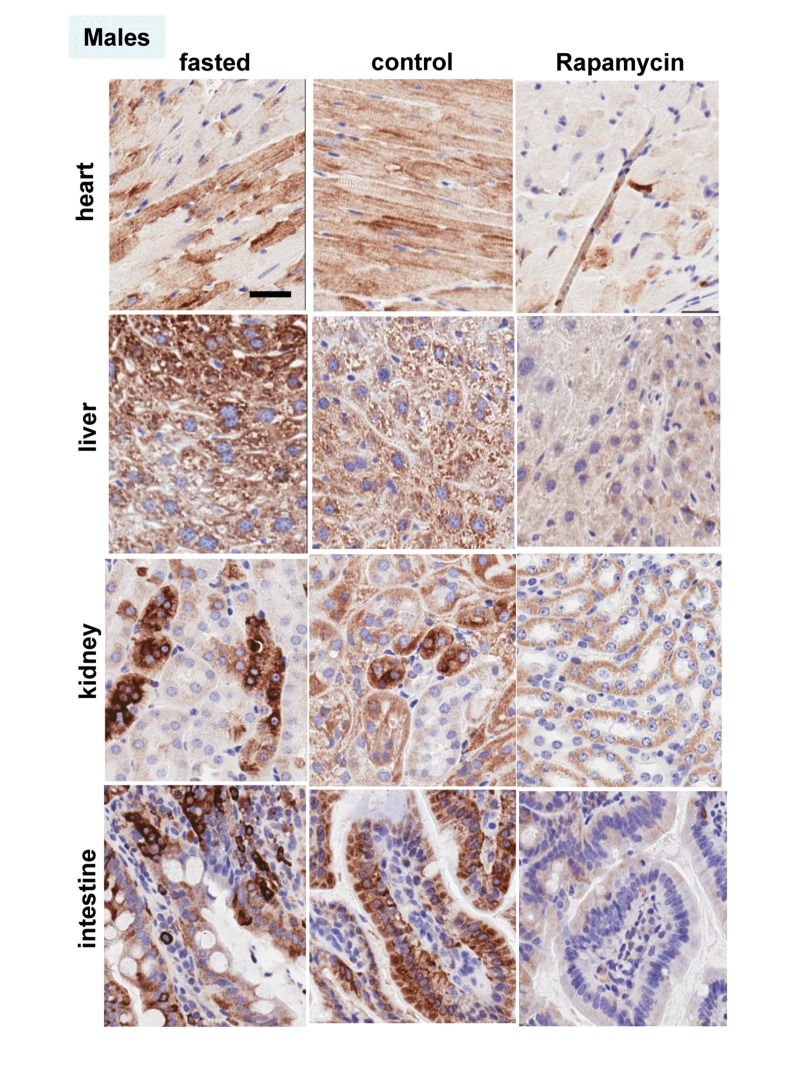
Males: Immunochemistry of pS6 in the organs pS6 in different organs of males (~ 10 months old). Fasted - mice were fasted overnight and sacrificed; control – mice received food ad libitum; rapamycin – mice received 1.5 mg/kg rapamycin i.p. and sacrificed 1 h later. Bar – 30 μm.

These results were confirmed in four organs (heart, liver, intestine, kidney) performed by immuno-histochemistry (Fig. 6, 7). First, there was no significant difference between fasted and fed ad libitum animals, indicating that fasting just marginally inhibited pS6. In contrast, in rapamycin-treated mice, levels of pS6 were dramatically decreased in all 4 organs (Fig. 6, 7).

However, the effect of rapamycin was more pronounced in females compared to males (Fig. 6 vs 7). In males, rapamycin decreased levels of S6 phosphorylation but pS6 staining was still well detectable in all 4 organs (Fig. 7). However, in female organs rapamycin not only decreased levels of pS6 but also completely eliminated it, making it undetectable (Fig. 5A and 6).

## DISCUSSION

Why males, who are robust in young age, usually undergo unhealthy and rapid aging and die relatively fast? There are many explanations including unhealthy life style, at least, in humans. But the universality of the phenomenon is startling. Among hundreds of theories there were some that propose that women live longer after menopause for the purpose to help their daughters to raise grandchildren [137-139]. Such theories imply that diseases like menopause may be programmed. In contrast, menopause is an age-related disease because it increases mortality [140]. Like aging and diseases, menopause is not programmed but quasi-programmed [12]. Still females live longer. Females compete to a lesser extent and their accidental death rate is lower than in young males. Therefore, they are often smaller and weaker than males. Taking this into consideration, hyperfunction theory allowed us to solve the mystery of female longevity. Young females, which are smaller than males, might have a lower activity of the mTOR pathway. Since phosphorylation of S6 was inhibited by rapamycin, we consider it as the most reliable marker of mTORC1 activity, which is rapamycin sensitive. Here we assessed the activity of the mTOR pathway by pS6 (a marker of mTORC1) and p-AKT (Ser 473), which is a rapamycin-insensitive marker of mTORC2. At age of 6 months, males were significantly heavier than females. Both pS6 and p-AKT were statistically higher in at least some male organs, as indicated by both immunoblotting and immunohistochemistry (Fig. 2 and 3). Levels of pS6 significantly correlated with body weight and p-AKT (Fig. 2 and 3). Male mice had significantly higher fasted insulin levels and higher insulin response to re-feeding when compared to females (Fig. 1D and E). From 6 to 10 months age, the difference between males and females become less prominent. Since levels of pS6 were variable among individual mice, fasting decreased the average pS6 only marginally (control and fasted mice are different individuals) and a larger study may be required. In contrast to fasting, intraperitoneal (i.p.) administration of rapamycin dramatically decreased pS6 in all organs tested (the liver, the heart, the intestine, the kidney) (Fig. 4A, 5A, 6 and 7). The magnitude of S6 dephosphorylation by rapamyin was more pronounced in females than in males as seen on overexposed immunoblot of liver samples (Fig. 5A) and on immuno-histochemically stained slides of four organs (Fig. 6 and 7). In conclusion, our study demonstrated that both p-AKT and pS6 were higher in young males than in young females. These data can explain robust growth and faster aging in male species. Higher sensitivity of females to rapamycin explains superb life extension observed in females compared to males in several studies [86-88].

Many questions remain. Is p-AKT predominantly a marker of mTORC2 activity or also a marker of the activity of the AKT/mTOR pathway, acting upstream of mTOR. What other kinases are involved in S6 phosphorylation? What are other kinase pathways involved in senescence? What are other sites of phosphorylation and modification of multiple components of the mTOR pathway (including Akt, AMPK, TSC, IRS, PI-3K, elongation factors, raptor, TOR itself and so on) could be biomarkers of longevity, health and aging? How does the activity of the MTOR pathway affect the difference in longevity among species? For millennia, people erroneously thought that biological aging is caused by accumulation of all sorts of damage, a process similar to the decay of the Egypt pyramids or car rusting. More recently aging has been believed to be caused by accumulation of molecular damage such as DNA damage by free radicals [141-154] but this has been practically disproved [113, 155-168]. Also, mild hormesis that increases life span induces molecular damage [169-172].

Consider the simplest paradox of the molecular theory of aging. Obviously, nutrients provide energy to repair molecular damage. If this damage is a cause of aging, animals would live longer. However, it is exactly opposite: nutrients and obesity accelerate aging, whereas calorie restriction increases lifespan [55, 148, 149, 173-188]. It is possible to speculate about a mysterious allocation of energy to the anti-aging repair during famine and starvation (perhaps we can even doubt the law of energy conservation). But the fact is: the more energy (nutrients), the shorter lifespan. But the very simple solution to this paradox is that aging is not caused by accumulation of molecular damage [189]. Instead, aging is driven by the mTOR pathway, which is activated by growth factors, nutrients (food), insulin (which is induced by nutrients), testosterone and some other factors that all stimulate cellular and organismal growth. When development is completed the same still active mTOR pathway then drives aging and age-related diseases [189]. In other words, aging is a quasi-program of development, an aimless continuation of growth driven by nutrient/insulin-sensing signaling pathways [60]. Therefore, males live shorter not because they are too weak but because they are too robust (due to hyperactive mTOR).

Our work complements outstanding discoveries by Bartke and co-workers that high levels of growth hormone shorten life span. In fact, high levels of growth hormone (GH), IGF-1, insulin all decrease lifespan in mice. Such mice are big and short-lived. In contrast, mice deficient in GH/IGF-1 signaling live longer [190-198]. The GH/IGF-1 axis activates the mTOR pathway. In line with the hyperfunction theory is an excellent observation that big mice die young: early life body weight predicts longevity in genetically heterogeneous mice [199]. Our data provide mechanistic explanation: the higher the TOR activity, the bigger the mice. Early life growth hormone treatment shortens longevity [200, 201] and instead antagonists of these hormones may extend life span, treat cancer and some age-related diseases [202-206].

TOR-driven cellular hyperfunction and aging cause organ damage. For example, cancer cells are not weak but instead robust despite high levels of damage including DNA damage. An organism dies because cancer cells are too robust not because they are weak due to damage. Robustness, in part, can be explained by hyperactive TOR in cancer cells, which is the most common alteration in cancer and a target for therapy [125, 136, 207-239].

The hyperfunction theory suggests that if aggressive males must have high mTOR activity early in life, they must age fast too. Furthermore, the theory suggests that these aging-promoting genes like mTOR are antagonistically pleiotropic [110]. Active and robust mTOR pathway provides advantage early in life including reproduction (noteworthy, mTOR increases spermatogenesis and fertility). At the same time mTOR decreases survival much later in life, when an organism would not exist in dangerous for males natural environment anyway. And this mechanism is much more important to males than to females. It was theoretically described why and how males must age faster and die younger than females [12]. However, for the first time this hypothesis was supported experimentally.

## MATERIALS AND METHODS

### Mice

All animal studies were conducted in accordance with the regulations of the Committee of Animal Care and Use at Roswell Park Cancer Institute. Mice were kept in polypropylene cages (30 × 21 × 10 cm) under standard light/dark regimen (12 hours light: 12 hours darkness) at 22 ± 2 °C and received standard laboratory chow (5% fat).

### Study in 6 months old mice

Mice of C57BL/6NCr strain, 6 months old, were divided into 3 groups: 10 females and 10 males were fasted overnight and 10 females were fed ad libitum. Fasted and non-fasted blood sera were prepared, accordingly, for biochemical analysis. Fasted animals were re-fed and sacrificed 2 hour later.

### Study in 10 months old mice

16 females and 18 males of C57BL/6NCr strain were randomly assigned to 3 of the following groups: fasted, control (received food ad libitum) and rapamycin treated. Mice in rapamycin group received rapamycin (purchased from LC laboratories, USA) at 1.5 mg/kg intraperitoneally (i.p.) and sacrificed an hour later. Mice in fasted group were fasted overnight and sacrificed. Non-fasted (fed) and fasted blood was collected. Blood was also collected after re-feeding during sacrifice. Glucose levels were measured directly in blood upon collection using Accu-Chek Aviva strips (McKesson, Atlanta, GA). Sera were prepared and used for biochemical analyses.

### Insulin concentration in blood sera

Was measured using Insulin (Mouse) Ultrasensitive ELISA kit (ALPCO Diagnostics, Salem, NH) according to manufacture's protocol. Data were analyzed using range of insulin standards and four parameter logistic fit.

### Statistical analyses

*T* test and correlation analyses (Pearson *r* coefficient and *p* value (two tailed)) were performed using GraphPad Prism version 5.00 for Windows, GraphPad Software, San Diego California USA, www.graphpad.com.

### Western blot analysis

Tissues were homogenized in a Bullet blender using stainless steel 0.5 mm diameter beads (Next Advantage, Inc, Averill Park, NY, USA) and RIPA lysis buffer supplemented with protease and phosphatase inhibitor tablets (Roche Diagnostics, Indianapolis, IN, USA). Lysates were cleared by centrifugation at 4°C at 13,000 rpm. Equal amounts of protein were separated on gradient Criterion gels (BioRad) and immunoblotting was performed with rabbit anti-phospho S6(Ser 240/244), anti-phospho AKT (Ser473), anti-AKT and mouse anti-S6 antibodies from Cell Signaling Biotechnology as described previously [36, 39, 40]. Rabbit anti-actin antibody was from Sigma-Aldrich. Secondary antibodies were from Cell Signaling.

### Immunochemistry

Dissected tissue samples were fixed in 10% buffered formalin, embedded into paraffin. 4 μm thin histological sections were stained with anti-phospho S6 (Ser240/244) antibody (Cell Signaling), followed by biotinylated goat-anti-rabbit secondary antibody (Vector, cat # BA-1000, Burlingame, CA) and counterstained with Hematoxylin.

Conflict of Interest Statement The authors of this manuscript have no conflict of interests to declare.

## SUPPLEMENTAL FIGURE


