# Supplementary material for: Mechanistic or mammalian target of rapamycin (mTOR) may determine robustness in young male mice at the cost of accelerated aging
Source: Aging (Albany NY). 2012 Dec 21;4(12):899–916. doi: 10.18632/aging.100528 (PMC3615157; doi:10.18632/aging.100528)
Supplement: Supplementary file 1 [file aging-04-899-s001.pdf]

SUPPLEMENTAL FIGURE

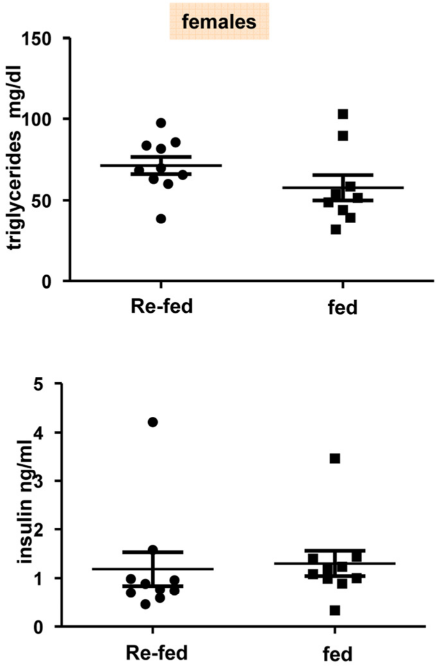

Figure S1. Levels of insulin and triglycerides in two sub-groups of females: individual mice 1-10 versus individual mice 21-30.
